# Comparison of learning needs priorities between healthcare providers and patients with heart failure

**DOI:** 10.1371/journal.pone.0239656

**Published:** 2020-09-24

**Authors:** Deulle Min, Jin-Sun Park, Eui-Young Choi, Jeong-Ah Ahn

**Affiliations:** 1 Department of Nursing, College of Medicine, Wonkwang University, Iksan, Korea; 2 Department of Cardiology, Ajou University School of Medicine, Suwon, Korea; 3 Division of Cardiology, Gangnam Severance Hospital, Yonsei University College of Medicine, Seoul, Korea; 4 College of Nursing and Research Institute of Nursing Science, Ajou University, Suwon, Korea; University of Adelaide, AUSTRALIA

## Abstract

It is necessary to understand the learning needs of heart failure (HF) patients to provide adequate patient education. It is necessary to identify what HF patients want to know and how this differs from the educational needs of healthcare providers. The aim of this descriptive and exploratory study was to evaluate and compare the learning needs priorities between HF patients and their healthcare providers. One hundred patients with HF and 20 healthcare providers were recruited from cardiovascular outpatient clinics at 2 large tertiary medical centers in South Korea. Learning needs were measured using a self-administered questionnaire with the Heart Failure Patients’ Learning Needs Inventory. Data were analyzed using SPSS 23.0 program. Overall rank orders for 48 items were similar in both groups (Spearman rank order correlation 0.605, *p* < .001). The educational topics of medications and worsening signs and symptoms ranked highest in both groups. However, healthcare providers were more concerned with diet management than were the patients (mean score 4.18 *vs*. 3.62; *p* = .001). The study showed both similarities and differences between the assessments of the patients and healthcare providers of detailed educational learning needs. It is important to develop patient-centered educational materials considering HF patients’ actual learning needs, and also to provide comprehensive and practical patient education based on a supportive understanding of healthcare provider needs.

## Introduction

The prevalence of heart failure (HF) is continuously increasing in accordance with the global expansion of elderly populations with increased risk factors for hypertension and coronary heart disease [1]. It affects approximately 26 million individuals worldwide, with estimated health expenditures of approximately $31 billion per year in the United States, which are projected to increase by approximately three-fold by 2030 [2–4]. A major portion of the burden in HF is that currently available treatment options are only focused on relieving patient symptoms, and there is a lack of ultimately curative treatments for the disease [5]. Furthermore, hospitalization and readmission rates for HF continue to increase [6], with more than 20% of HF patients readmitted within 30 days and up to 50% by 6 months [7].

HF is associated with various risk factors including older age, obesity, smoking, hypertension, hyperlipidemia, diabetes, and previous cardiac ischemia or dysrhythmia [8, 9]. The major guidelines addressing HF are targeted to manage the underlying mechanisms affected by the disease process and to monitor patient clinical signs and symptoms [10]. Therefore, it is important to help patients understand their disease process with appropriate healthcare information and to ensure their proper monitoring as part of lifelong, ongoing healthcare for which comprehensive patient education, including medications, exercise, diet and daily lifestyle changes, is necessary [11]. As such, determining patient learning needs should be addressed as early as possible to provide the most effective education [12].

In the past, patient education was determined based on the healthcare provider’s perspective. However, it has recently been emphasized that considering patient perspective and partnership plays an important and key role in patient education [13]. Patient-centered education is a partnership among patients, families, and healthcare providers, and it can promote patient satisfaction with education and successful implementation, and further improve disease and health outcomes [14, 15].

Several previous studies have investigated the learning needs of HF patients [16–18]. However, to date, there is a lack of studies evaluating differences between HF patients’ and their healthcare providers’ perspectives and opinions regarding patient education.

Therefore, the aim of the present study was to identify the learning needs of HF patients and to recognize information gaps by comparing the learning needs priorities between HF patients and their healthcare providers.

## Materials and methods

### Study design

The present investigation was a descriptive and exploratory study to investigate and compare the learning needs priorities between patients with HF and their healthcare providers.

### Setting and samples

One hundred patients with HF and 20 healthcare providers (15 nurses, 5 physicians) were recruited through convenience sampling from cardiovascular outpatient clinics at 2 large tertiary medical centers in Seoul and Suwon city, Korea. Data were collected from June to October 2017. Patients were included if they were adults over 18 years of age, diagnosed with HF by a cardiologist and regularly visiting for medical follow-ups, able to communicate, and willing to participate in the study. Healthcare providers were included if they were nurses or physicians with clinical careers more than 5 years in the same cardiovascular clinics as the patients.

### Measurements

Participants completed a self-administered questionnaire surveying general and disease-related characteristics and educational learning needs. Learning needs were measured using the Heart Failure Patients’ Learning Needs Inventory (HFPLNI), developed by Wehby and Brenner [19] and translated into Korean by Kim et al. [20]. The HFPLNI consists of 48 questions with 8 subscales: general HF information; psychological information; risk factors; medications; diet; activity; prognosis, and signs and symptoms. Each item is scored using 5-point Likert scale, with a higher score indicating a higher level of importance. The overall Cronbach’s alpha was 0.96 [19] and 0.97 [20] in previous studies, and 0.98 in the present study.

### Data analyses

Data were analyzed using SPSS version 23.0 (IBM Corporation, Armonk, NY, USA). Descriptive statistics were used to explain general and disease-related characteristics, and the levels of learning needs of HF patients and healthcare providers. Kolmogorov-Smirnov and Shapiro-Wilk tests indicated that both HF patients’ and healthcare providers’ data used were normally distributed (*p* > .05). Mean scores obtained for each question were ranked from the best to the worst score within each group. The Spearman’s rank correlation test was used to identify the relationship between the patients’ and healthcare providers’ responses. Independent sample t-tests were performed to compare learning needs between HF patients and healthcare providers.

### Ethical considerations

This study was approved by the Institutional Review Boards of Ajou University Hospital and Gangnam Severance Hospital, Yonsei University before initiation of the study (IRB no. AJIRB-MED-SUR-17-179 & 3-2017-0088). All participants provided written informed consent and were assured that their information would remain confidential. Participant agreement forms were obtained after the researcher explained the purpose of the study and confidentiality of the data. Anonymity was guaranteed by suppressed encoding of each participant’s name.

## Results

### General and disease-related characteristics of participants

General and disease-related characteristics of patients with HF are presented in Table 1. The mean age of the patients was 72.15 years, and 58% were women. Fifty-two percent of the patients had more than a high school education level, 76% reported higher than middle socioeconomic status, and 75% reported no occupation. Patients were diagnosed with HF for an average of 6.16 years, 49.0% of whom reported New York Heart Association (NYHA) functional class I-II, and 51.0% reported NYHA class III-IV. Most patients (95%) were currently taking regular medication.

**Table 1:** General and disease-related characteristics of patients with heart failure.

Characteristics	Mean ± SD or *n* (%)
Age (range: 44–94 years)	72.15 ± 11.08
Gender	
Men	42 (42.0)
Women	58 (58.0)
Educational level	
Middle school or less	48 (48.0)
High school or above	52 (52.0)
Socioeconomic status	
Low	24 (24.0)
Middle	57 (57.0)
High	19 (19.0)
Marital status	
Married	74 (74.0)
Widowed	24 (24.0)
Divorced	2 (2.0)
Employment status	
Yes	25 (25.0)
No	75 (75.0)
Religion	
Christianity	34 (34.0)
Buddhism	23 (23.0)
Catholicism	11 (11.0)
None	32 (32.0)
Duration of heart failure (years)	6.16 ± 5.49
NYHA^a^ class	
I	24 (24.0)
II	25 (25.0)
III	35 (35.0)
IV	16 (16.0)
Treatment^b^	
Medication therapy	95 (95.0%)
Internal intervention	23 (23.0%)
Surgical operation	17 (17.0%)
Family history of cardiovascular disease	
Yes	19 (19.0)
No	81 (81.0)

^a^New York Heart Association.

^b^Overlapping response.

General characteristics of healthcare providers are presented in Table 2. The mean age of this group was 36.8 years, and 75% were women. The mean length of healthcare providers’ clinical career in the cardiovascular division was 8.88 years, and 30.0% had a Master’s or Doctoral degree.

**Table 2:** General characteristics of healthcare providers.

Characteristics	Mean ± SD or *n* (%)
Age (years)	36.80 ± 9.38
Gender	
Men	5 (25.0)
Women	15 (75.0)
Occupation	
Nurse	15 (75.0)
Physician	5 (25.0)
Clinical career in cardiovascular division (years)	8.88 ± 6.74
Educational level	
Undergraduate	14 (70.0)
Graduate	6 (30.0)

### Comparison of learning needs of HF patients and healthcare providers according to educational topics

The total mean scores of learning needs of patients with HF and healthcare providers were 3.76/5 and 3.98/5 points, respectively. The two educational topics ranked highest by the patients were also the two ranked highest by healthcare providers-namely, “Medications” and “Signs and symptoms”. The educational topic of “Psychological information” was ranked the lowest by both groups (Table 3).

**Table 3:** Comparison of learning needs of patients and healthcare providers according to educational topics.

Educational topics	Heart failure patients		Healthcare providers		t	*P*
	(n = 100)		(n = 20)			
	Mean ± SD	Rank	Mean ± SD	Rank		
General heart failure information	3.72 ± 0.89	5	4.07 ± 0.67	4	1.662	.099
Psychological information	3.49 ± 1.02	8	3.60 ± 0.99	8	0.441	.660
Risk factors	3.82 ± 1.11	3	3.77 ± 0.91	7	0.215	.831
Medications	4.06 ± 1.09	1	4.20 ± 0.67	2	-0.767	.447
Diet	3.62 ± 0.95	6	4.18 ± 0.59	3	3.454	.001
Activity	3.56 ± 0.97	7	3.85 ± 0.58	6	1.804	.078
Prognosis	3.80 ± 0.93	4	3.88 ± 0.86	5	-0.370	.712
Signs and symptoms	4.04 ± 1.01	2	4.26 ± 0.55	1	1.362	.179
Total	3.76 ± 0.82		3.98 ± 0.58		1.383	.175

Ranks are presented from the best to the worst score.

When comparing the mean scores of learning need topics, the scores related to “Diet” were significantly different between HF patients and healthcare providers (3.62 *vs*. 4.18, respectively; *p* = .001) (Table 3).

### Comparison of learning needs priorities between HF patients and healthcare providers according to questionnaire items

Detailed rank orders of importance according to questionnaire items between HF patients and healthcare providers are presented in Table 4. The correlation between patients’ and healthcare providers’ learning needs was 0.605 (*p* < .001, Spearman test)-that is, the rank orders for the 48 items were similar.

**Table 4:** Comparison of learning needs priorities between patients and healthcare providers according to question items.

	Questionnaire items	Heart failure patients		Healthcare providers	
		(n = 100)		(n = 20)	
		Mean ± SD	Rank	Mean ± SD	Rank
	*I need to know (or teach)*				
Q 1	…why I am short of breath	3.96 ± 1.24	17	4.05 ± 1.00	23
Q 2	…what the heart looks like and how it works	3.41 ± 1.53	40	3.70 ± 1.26	40
Q 3	…what cause heart failure	3.89 ± 1.28	21	3.95 ± 1.10	27
Q 4	…what happens when someone has heart failure	4.04 ± 1.11	11	4.45 ± 0.95	3
Q 5	…can the heart’s function improve	3.96 ± 1.10	18	4.20 ± 0.83	17
Q 6	…if any other tests will be done after I leave the hospital	3.39 ± 1.35	41	3.85 ± 0.88	34
Q 7	…the reason for further testing after I go home	3.47 ± 1.35	38	4.30 ± 0.92	8
Q 8	…where my family can go to learn CPR	3.31 ± 1.44	44	3.85 ± 1.23	32
Q 9	…the normal emotional response to having a chronic illness	3.47 ± 1.31	39	3.40 ± 0.94	48
Q10	…the importance of talking to someone about my fears, feelings, and thoughts	3.35 ± 1.40	43	3.55 ± 1.23	43
Q11	…what effect stress has on my heart	3.89 ± 1.19	22	3.80 ± 1.15	35
Q12	…what I can do to reduce stress while in the hospital	3.60 ± 1.30	33	3.40 ± 1.14	45
Q13	…what I can do to reduce stress when I go home	3.69 ± 1.24	29	3.70 ± 1.17	41
Q14	…what support groups are available	2.94 ± 1.48	47	3.75 ± 1.02	38
Q15	…which factors may have contributed to the onset of my heart disease	3.66 ± 1.33	30	3.40 ± 1.10	46
Q16	…how these factors affect the heart	3.73 ± 1.30	27	3.65 ± 1.04	42
Q17	…what I can do to improve my heart function	4.08 ± 1.13	7	4.25 ± 1.02	10
Q18	…general rules about taking medications	4.01 ± 1.20	14	4.35 ± 0.93	5
Q19	…why I am taking each medications	4.03 ± 1.22	13	4.25 ±0.91	11
Q20	…what the side effects of each medications are	4.04 ± 1.29	10	4.15 ± 0.75	20
Q21	…what to do if I have problems with medications	4.16 ± 1.20	4	4.15 ± 0.88	19
Q22	…how to adapt to taking medications every day	4.05 ± 1.10	9	4.10 ± 0.91	22
Q23	…general rules about eating	3.72 ± 1.27	28	4.21 ± 0.71	15
Q24	…how diet affects my heart disease	3.86 ± 1.20	23	4.25 ± 0.72	13
Q25	…what the words sodium, salt, and NaCl mean	3.61 ± 1.39	32	4.05 ± 0.76	24
Q26	…what my diet restrictions are, if any	4.06 ± 1.13	8	4.20 ± 0.77	18
Q27	…how to adapt the recommended diet to my lifestyle	3.92 ± 1.15	19	4.20 ± 0.89	16
Q28	…what fluid restriction means	3.55 ± 1.23	35	4.30 ± 0.73	9
Q29	…how to adapt the recommended fluid restriction to my lifestyle	3.55 ± 1.25	34	4.25 ± 0.72	13
Q30	…why daily weights are needed	3.08 ± 1.32	45	4.10 ± 1.02	21
Q31	…how to adapt daily weights to my lifestyle	3.03 ± 1.38	46	3.90 ± 0.85	30
Q32	…how alcohol affects the heart	3.82 ± 1.27	25	4.40 ± 0.68	4
Q33	…why I may not be able to do as much physically as I could before developing heart failure	3.99 ± 1.17	15	3.90 ± 0.64	31
Q34	…general guidelines for physical activity	3.86 ± 1.14	24	3.95 ± 0.83	29
Q35	…what my physical activity restrictions are, if any	3.90 ± 1.22	20	4.05 ± 0.69	25
Q36	…how to tell if I can increase my activity	3.75 ± 1.22	26	3.80 ± 0.61	36
Q37	…when I can engage in sexual activity	2.32 ± 1.41	48	3.55 ± 0.89	44
Q38	…how significant my heart failure is	4.03 ± 1.23	12	4.35 ± 0.75	7
Q39	…what advanced directives are	3.49 ± 1.53	37	3.75 ± 1.21	37
Q40	…what is my long-term life expectancy	3.38 ± 1.43	42	3.40 ± 1.10	46
Q41	…what can happen if I do not follow my doctor’s recommendations	4.28 ± 1.07	1	4.25 ± 0.91	11
Q42	…what my quality of life is expected to be	3.65 ± 1.17	31	3.85 ± 1.04	33
Q43	…what advice should be given to my family in the event of a sudden death outside the hospital	3.98 ± 1.26	16	3.70 ± 1.38	39
Q44	…what symptoms are caused by heart failure	4.10 ± 1.16	6	4.35 ± 0.81	6
Q45	…what are the signs and symptoms of worsening heart failure	4.19 ± 1.14	3	4.50 ± 0.51	2
Q46	…what I should do if symptoms worsen	4.24 ± 1.07	2	4.50 ± 0.61	1
Q47	…when to call the doctor	4.15 ± 1.11	5	4.00 ± 1.12	26
Q48	…the signs and symptoms of other heart problems	3.54 ± 1.40	36	3.95 ± 0.89	28

Ranks are presented from the best to the worst score.

The item ranked the highest score by the patients was “What can happen if I do not follow the doctor’s recommendations?” (Q41), and it was ranked 11^th^ by healthcare providers. The next two items ranked (2^nd^ and 3^rd^) highest by the patients were the two ranked (1^st^ and 2^nd^) highest by healthcare providers-namely, “What are the signs and symptoms of worsening HF?” (Q46) and “What should I do if symptoms worsen?” (Q45).

In contrast, the item ranked the lowest score (48^th^) by the patients was “When can I engage in sexual activity?” (Q37), and it was ranked 44^th^ by healthcare providers. The item ranked the lowest score (48^th^) by healthcare providers was “What is the normal emotional response to having a chronic illness?” (Q9), and it was ranked 39^th^ by the patients.

Items that demonstrated significant differences in rank between the two groups were as follows. “When to call the doctor” (Q47) and “Family education for the event of a sudden death” (Q43) were ranked relatively higher among the patients (5th and 16th), but relatively lower among healthcare providers (26th and 39th). On the other hand, the items related to “Reason for further testing” (Q7), “Fluid restriction” (Q28 and 29), “Control of daily weights” (Q30), and “Effect of alcohol” (Q32) demonstrated relatively lower ranking among the patients (25th to 45th); however, they demonstrated relatively greater importance among healthcare providers (4th to 21st).

## Discussion

This study aimed to investigate learning needs among patients with HF and to compare priorities with those of healthcare providers. Results showed both similarities and differences between the assessments of the patients and healthcare providers of the learning needs. Overall, the rank order of the learning needs’ evaluation was similar in the two groups analyzed.

Regarding educational topics, HF patients expressed a desire to be educated with a focus on their medications and worsening signs and symptoms. Healthcare providers also wanted to educate the most about the same topics as the patients. Our results are consistent with those from previous studies that examined the learning needs of patients with HF. Wehby and Brenner [19] investigated the learning needs of HF patients and nurses, and found that medications and signs and symptoms were relatively more demanded topics than other topics in both groups. Similarly, Ong et al. [21] reported that the most important learning need of hospitalized HF patients was the topic on signs and symptoms of HF, and Yu et al. [18] presented that the information HF patients most needed before being discharged from the hospital was about medication. Medication knowledge has a significant impact on HF patients’ medication adherence, which in turn has a significant impact on their health outcomes [22]. One of the major aims of HF management is to alleviate the symptoms of HF [23]. Therefore, proper education about medications and signs and symptoms are very important educational topics that cannot be overemphasized for both HF patients and healthcare providers.

In the present study, healthcare providers were more concerned with diet than the patients. The score related to the topic of diet was significantly higher in healthcare providers than in HF patients. This was similar to the finding of Ong et al. [21] that education topic related to diet was poorly valued by the HF patients. Furthermore, detailed items that HF patients and healthcare providers had different views were about fluid restriction, alcohol consumption, and daily weights among others in this study. However, these are considered to be essential aspects of self-management by HF patients [24, 25]. The main goals of HF management are to reverse or, at least mitigate the effects of cardiac dysfunction (e.g., venous congestion), and to reduce or eliminate factors that affect disease progression. In this regard, restrictions regarding intake of sodium and water are particularly emphasized to reduce venous congestion and related signs and symptoms [26]. It appears that dietary habits among HF patients are deeply ingrained, and they may be less interested in changing. Sometimes, HF patients complain more difficulty in adhering to lower fluid allotments with as few as 60% reporting adherence to the fluid restriction [27], and some may not be well-versed on the close relationship between fluid retention and clinical symptoms of HF. Although patients acknowledge the importance of diet, they should be helped with daily self-management [16]. In order to improve HF self-management, it is undoubtedly necessary for healthcare providers to investigate the level of knowledge of their patients before educating them and providing tailored interventions based on their actual learning needs. Although it is important to provide medical information and knowledge, it is also critical to consider how it will be best applied in the real-world lives of HF patients [28].

Although there was statistical similarity between the two groups in the overall rank order of the learning needs, we identified that the ranks of detailed items that HF patients and healthcare providers consider important were quite different from one another. Patients want to ask about their illness or treatments in the hospital; however, they feel persistent difficulties in communicating with their healthcare providers [29]. Sometimes, patients do not report all the problems because they are fearful of healthcare providers’ inappropriate reaction to the patients’ concerns, for instance, ignoring them [30]. In some cases, patients believe that healthcare providers give them too much or inappropriate information [31]. However, healthcare providers generally tend to perceive that patients do not fully understand what they must do [16]. Consequently, inappropriate communication can be a large barrier to effective management of HF patients [31, 32].

In a previous study, HF nurses’ tailored and individual education was shown to be effective in improving self-management ability of HF patients [33]. Patient-centered education in consideration of the patient’s priority learning needs can improve the patient’s confidence in self-management as well as satisfaction with patient-healthcare provider communication and overall clinical care [34]. In addition, a previous study presented that the individual education level or different disease condition of the patient can impact the patient-healthcare provider communication and further preferences on the patient-centered care [35]. Therefore, it is necessary to develop carefully tailored programs that can facilitate communication between HF patients and healthcare providers, considering the patients’ actual learning needs. Moreover, it is also necessary to develop patient-centered educational materials to provide practical education with thoughtful understanding of individual status and preferences.

This study has several limitations. First, small and convenience sample is not representative of the population and therefore has poor generalizability. Especially, the sample size for healthcare providers was very low, precluding comparison between nurses and doctors and potentially impacting the validity of the findings. It will be valuable to consider diverse healthcare professionals’ aspects in the future study. Second, the Cronbach's alpha of the HFPLNI questionnaire was 0.98 in this study, that was very high. It might make difficult to differentiate the priorities of each item as participants tended to give the same response to the most items. In future research, it is possible to get clearer rankings and priorities by either using a response scale with more than 5 points or letting the participants rank the different items.

## Conclusions

There were both similarities and significant gaps between the learning needs and priorities of HF patients and those of healthcare providers. Healthcare providers need to assess actual learning needs and the level of education of HF patients, and to provide individual patient-centered education in a comprehensive and practical manner to design effective strategies for successful self-management throughout a patient’s life.

## Supporting information

S1 File (SAV)
